# Novel Targeted Therapies for Advanced Cholangiocarcinoma

**DOI:** 10.3390/medicina57030212

**Published:** 2021-02-26

**Authors:** Alessandro Rizzo, Giovanni Brandi

**Affiliations:** 1 Department of Experimental, Diagnostic and Specialty Medicine, S. Orsola-Malpighi University Hospital, 40138 Bologna, Italy; giovanni.brandi@unibo.it; 2 Division of Oncology, IRCCS Azienda Ospedaliero-Universitaria di Bologna, 40138 Bologna, Italy

Cholangiocarcinoma (CCA) includes a group of rare and aggressive hepatobiliary malignancies, including extrahepatic cholangiocarcinoma (eCCA) and intrahepatic cholangiocarcinoma (iCCA), with the former further subdivided into distal (dCCA) and perihilar cholangiocarcinoma (pCCA) [1,2]. Notably enough, these subgroups not only arise from different anatomical locations of the biliary tree, but also present notable differences in terms of prognosis, etiology, biology, and epidemiology [3,4].

Over the last decade, the advent of next-generation sequencing has paved the way towards the identification of important molecular features of CCA, with a large number of reports observing genetic aberrations that are exclusive to specific CCA subtypes [5,6]. These findings have led to the development of several molecularly targeted therapies in this setting, with approximately 50% of CCA patients harboring potentially druggable aberrations [7,8]. In fact, a number of potential therapeutic targets have been described, including fibroblast growth factor receptor (FGFR) fusions, mutations in isocitrate dehydrogenase (IDH)-1, BRAF mutations, and neurotrophic tyrosine kinase (NTRK) gene fusions [9,10,11,12].

As regards FGFR targeted agents, the FGFR1, FGFR2, and FGFR3 inhibitor pemigatinib received Food and Drug Administration (FDA) approval in April 2020 for previously treated CCA patients harboring FGFR2 fusions or rearrangements [13,14,15]. The approval was based on the results of the phase II FIGHT-202 clinical trial, where pemigatinib reported an overall response rate (ORR) of 35% and a median overall survival (OS) of 21.1 months, after a median follow-up of 17.8 months [16]. In addition, several other FGFR inhibitors are being assessed and are currently in different stages of development in CCA patients, including derazantinib, infigratinib, and futibatinib, with the latter recently showing an ORR of 37.3% and median duration of response of 8.3 months in the FOENIX-CCA2 clinical trial [17,18,19].

Similarly, IDH inhibitors are being investigated in CCA, with IDH-1 mutations occurring in approximately 13–15% of iCCA patients [20]. The recently published ClarIDHy phase III trial compared the IDH-1 inhibitor ivosidenib versus placebo in IDH-1 mutant CCA who had received up to two lines of systemic treatment [21]. Notably enough, the ivosidenib arm showed improved progression-free survival (PFS) compared to the placebo group, with median PFS of 2.7 months and 1.4 months, respectively (Hazard Ratio (HR) 0.37; 95% confidence interval (CI) 0.25–0.54; one-sided *p* < 0.0001) as well as a trend towards superior OS.

In addition, several other molecularly targeted treatments have recently reported interesting results, as witnessed by the findings of the phase II ROAR trial assessing the combination of dabrafenib plus trametinib in patients with BRAF^V600E^-mutated CCA [22,23]; moreover, an impressive number of phase I to III clinical trials are evaluating novel targeted therapies, as monotherapy or in combination with other anticancer agents, and might further modify the therapeutic landscape of CCA in the next years [24,25].

However, important issues remain to be addressed. First, the efficacy of targeted treatments is considerably limited by the onset on acquired resistance, with secondary polyclonal mutations representing a notable challenge in this setting [26,27]. Thus, the CCA medical community is showing growing interest towards the use of liquid biopsy, since this tool has the potential to track the emerging of polyclonal mutations and to guide treatment selection [28]. Second, combination therapies are being explored, with the aim of producing a synergist effect [29,30]. Third, the lack of predictive biomarkers able to guide therapeutic choices represents another important unmet need in this setting.

This Special Issue aims at highlighting several key open questions in CCA management and future perspectives for patients with advanced CCA, including novel targeted therapies, liquid biopsy, experimental treatments, and potential predictive biomarkers.

## References

[1] Razumilava N., Gores G.J. (2013). Classification, diagnosis, and management of cholangiocarcinoma. Clin. Gastroenterol. Hepatol..

[2] Rizzo A., Brandi G. (2020). BILCAP trial and adjuvant capecitabine in resectable biliary tract cancer: Reflections on a standard of care. Expert Rev. Gastroenterol. Hepatol..

[3] Banales J.M., Cardinale V., Carpino G., Marzioni M., Andersen J.B., Invernizzi P., Lind G.E., Folseraas T., Forbes S.J., Fouassier L. (2016). Cholangiocarcinoma: Current knowledge and future perspectives consensus statement from the European Network for the Study of Cholangiocarcinoma (ENS-CCA). Nat. Rev. Gastroenterol. Hepatol..

[4] Rizvi S., Gores G.J. (2017). Emerging molecular therapeutic targets for cholangiocarcinoma. J. Hepatol..

[5] Lamarca A., Barriuso J., McNamara M.G., Valle J.W. (2020). Molecular targeted therapies: Ready for “prime time” in biliary tract cancer. J. Hepatol..

[6] Massa A., Varamo C., Vita F., Tavolari S., Peraldo-Neia C., Brandi G., Rizzo A., Cavalloni G., Aglietta M. (2020). Evolution of the Experimental Models of Cholangiocarcinoma. Cancers.

[7] Ricci A.D., Rizzo A., Brandi G. (2020). Immunotherapy in Biliary Tract Cancer: Worthy of a Second Look. Cancer Control..

[8] Valle J.W., Lamarca A., Goyal L., Barriuso J., Zhu A.X. (2017). New Horizons for Precision Medicine in Biliary Tract Cancers. Cancer Discov..

[9] Churi C.R., Shro_ R., Wang Y., Rashid A., Kang H.C., Weatherly J., Zuo M., Zinner R., Hong D., Meric-Bernstam F. (2014). Mutation Profiling in Cholangiocarcinoma: Prognostic and Therapeutic Implications. PLoS ONE.

[10] Tella S.H., Kommalapati A., Borad M.J., Mahipal A. (2020). Second-line therapies in advanced biliary tract cancers. Lancet Oncol..

[11] Ricci A.D., Rizzo A., Brandi G. (2020). The DNA damage repair (DDR) pathway in biliary tract cancer (BTC): A new Pandora’s box?. ESMO Open..

[12] Tariq N.U., McNamara M.G., Valle J.W. (2019). Biliary tract cancers: Current knowledge, clinical candidates and future challenges. Cancer Manag. Res..

[13] Chakrabarti S., Kamgar M., Mahipal A. (2020). Targeted Therapies in Advanced Biliary Tract Cancer: An Evolving Paradigm. Cancers.

[14] Mertens J.C., Rizvi S., Gores G.J. (2018). Targeting cholangiocarcinoma. Biochim. Biophys. Acta Mol. Basis Dis..

[15] Morizane C., Ueno M., Ikeda M., Okusaka T., Ishii H., Furuse J. (2018). New developments in systemic therapy for advanced biliary tract cancer. Jpn. J. Clin. Oncol..

[16] Abou-Alfa G.K., Sahai V., Hollebecque A., Vaccaro G., Melisi D., Al-Rajabi R., Paulson A.S., Borad M.J., Gallinson D., Murphy A.G. (2020). Pemigatinib for previously treated, locally advanced or metastatic cholangiocarcinoma: A multicentre, open-label, phase 2 study. Lancet Oncol..

[17] Rizzo A., Ricci A.D., Brandi G. (2020). Futibatinib, an investigational agent for the treatment of intrahepatic cholangiocarcinoma: Evidence to date and future perspectives. Expert Opin. Investig. Drugs.

[18] Mazzaferro V., El-Rayes B.F., Droz dit Busset M., Cotsoglou C., Harris W.P., Damjanov N., Masi G., Rimassa L., Personeni N., Braiteh F. (2019). Derazantinib (ARQ 087) in advanced or inoperable FGFR2 gene fusion-positive intrahepatic cholangiocarcinoma. Br. J. Cancer.

[19] Botrus G., Raman P., Oliver T., Bekaii-Saab T. (2021). Infigratinib (BGJ398): An investigational agent for the treatment of FGFR-altered intrahepatic cholangiocarcinoma. Expert Opin. Investig. Drugs.

[20] Ma B., Meng H., Tian Y., Wang Y., Song T., Zhang T., Wu Q., Cui Y., Li H., Zhang W. (2020). Distinct clinical and prognostic implication of IDH1/2 mutation and other most frequent mutations in large duct and small duct subtypes of intrahepatic cholangiocarcinoma. BMC Cancer.

[21] Abou-Alfa G.K., Macarulla T., Javle M.M., Kelley R.K., Lubner S.J., Adeva J., Cleary J.M., Catenacci D.V., Borad M.J., Bridgewater J. (2020). Ivosidenib in IDH1-mutant, chemotherapy-refractory cholangiocarcinoma (ClarIDHy): A multicentre, randomised, double-blind, placebo-controlled, phase 3 study. Lancet Oncol..

[22] Rizzo A., Ricci A.D., Brandi G. (2020). Combination therapy of dabrafenib plus trametinib in patients with BRAF^V600E^-mutated biliary tract cancer. Hepatobiliary Pancreat. Dis. Int..

[23] Subbiah V., Lassen U., Élez E., Italiano A., Curigliano G., Javle M., de Braud F., Prager G.W., Greil R., Stein A. (2020). Dabrafenib plus trametinib in patients with BRAF^V600E^-mutated biliary tract cancer (ROAR): A phase 2, open-label, single-arm, multicentre basket trial. Lancet Oncol..

[24] Rizzo A., Ricci A.D., Brandi G. (2021). Recent advances of immunotherapy for biliary tract cancer. Expert Rev. Gastroenterol. Hepatol..

[25] Vogel A., Bathon M., Saborowski A. (2020). Immunotherapies in clinical development for biliary tract cancer. Expert Opin. Investig. Drugs.

[26] Saborowski A., Lehmann U., Vogel A. (2020). FGFR inhibitors in cholangiocarcinoma: What’s now and what’s next?. Ther. Adv. Med. Oncol..

[27] Goyal L., Shi L., Liu L.Y., Fece de la Cruz F., Lennerz J.K., Raghavan S., Leschiner I., Elagina L., Siravegna G., Ng R.W.S. (2019). TAS-120 Overcomes Resistance to ATP-Competitive FGFR Inhibitors in Patients with FGFR2 Fusion-Positive Intrahepatic Cholangiocarcinoma. Cancer Discov..

[28] Rizzo A., Ricci A.D., Tavolari S., Brandi G. (2020). Circulating Tumor DNA in Biliary Tract Cancer: Current Evidence and Future Perspectives. Cancer Genom. Proteom..

[29] Sipra Q.U.A.R., Shroff R. (2020). The impact of molecular profiling on cholangiocarcinoma clinical trials and experimental drugs. Expert Opin. Investig. Drugs.

[30] Rizzo A., Ricci A.D., Brandi G. (2021). PD-L1, TMB, MSI, and Other Predictors of Response to Immune Checkpoint Inhibitors in Biliary Tract Cancer. Cancers.

